# Deep brain stimulation of the nucleus basalis of Meynert in neurodegenerative diseases with cognitive impairment: An update on evidence and mechanisms

**DOI:** 10.4103/NRR.NRR-D-24-00838

**Published:** 2025-02-24

**Authors:** Xuyang Liu, Kai Shu, Liwu Jiao, Yumei Geng, Mengying Wang, Huicong Kang

**Affiliations:** 1 Department of Neurosurgery, Tongji Hospital, Tongji Medical College, Huazhong University of Science and Technology, Wuhan, Hubei Province, China; 2 Department of Neurology, Tongji Hospital, Tongji Medical College, Huazhong University of Science and Technology, Wuhan, Hubei Province, China

**Keywords:** Alzheimer’s disease, cholinergic pathway, cognition, deep brain stimulation, dementia, mechanism, neucleus basalis of Meynert, neurodegenerative diseases, neuromodulation, Parkinson’s disease

## Abstract

Current pharmacotherapy for neurodegenerative diseases is limited to providing symptomatic relief, instead of slowing or reversing disease progression. As a form of neuromodulation surgery, deep brain stimulation delivers electrical pulses through implanted electrodes in targeted brain regions and has been used to alleviate symptoms in neurodegenerative diseases. Depending on the precise targeting of neural modulation, deep brain stimulation is being explored for its potential to manage symptoms and improve overall quality of life in neurodegenerative diseases associated with cognitive impairment, such as Alzheimer’s disease and dementia in Parkinson’s disease. The nucleus basalis of Meynert, a critical component of the cerebral cholinergic system and the Papez circuit, is considered as a promising target for treating cognitive dysfunction in neurodegenerative diseases due to its essential role in regulating cognition, memory, and attention. However, the comprehensive mechanisms by which deep brain stimulation of nucleus basalis of Meynert affects neurodegenerative diseases with cognitive impairment remain largely uncharacterized. Nonetheless, various hypotheses and evidence from animal and clinical studies suggest mechanisms such as the modeulation of the cholinergic system, increased glucose metabolism and regional cerebral blood flow, neuroprotective effects, and the modulation of neural networks. In this review, we update the advances in research regarding the therapeutic effects and potential mechanisms of deep brain stimulation of nucleus basalis of Meynert on cognitive impairment in neurodegenerative diseases. Additionally, we examine the anatomy, connectivity, and physiological functions of the nucleus basalis of Meynert. Deep brain stimulation of nucleus basalis of Meynert may improve cognitive impairment in neurodegenerative diseases through multiple mechanisms; however, further larger-scale, multi-center clinical trials conducted at earlier disease stages are necessary to fully confirm its efficacy and safety.

## Introduction

Neurodegenerative diseases (NDDs) comprise a group of chronic, progressive, and incurable disorders such as Alzheimer’s disease (AD), Parkinson’s disease (PD), and Huntington’s disease (Izquierdo-Altarejos et al., 2024). Despite significant breakthroughs in the understanding of the pathophysiology of specific NDDs, such as the loss of dopaminergic neurons in PD and amyloid-β (Aβ) aggregation and tau protein hyperphosphorylation in AD, effective pharmacotherapy capable of delaying or even reversing the progression of these diseases is still lacking. This drawback, along with the confirmed widespread but specific neural network dysfunction in NDDs, has encouraged the adoption of deep brain stimulation (DBS) as a potential neuromodulatory and disease-modifying therapeutic approach for such conditions.

DBS is a stereotactic neuromodulatory surgical procedure whose use has been widely investigated in various neurological and neuropsychiatric disorders (Lee et al., 2019a; Limousin and Foltynie, 2019; Lozano et al., 2019; La Torre et al., 2020) and has been approved by the U.S. Food and Drug Administration for the treatment of PD, essential tremor, dystonia, epilepsy, and obsessive-compulsive disorder (Fasano et al., 2015; Moro et al., 2017; Krauss et al., 2021; Fisher, 2023; Sheth and Mayberg, 2023). An increasing number of animal studies and clinical trials have investigated the potential therapeutic effects and safety of DBS in PD and AD, with a particular focus on selecting targets with distinct neuromodulatory effects on specific dysfunctional neural circuits. Based on promising evidence of its impact on cognitive function, the effect of DBS on cognition-related structures and memory circuits, such as the nucleus basalis of Meynert (NBM), fornix, hippocampus, and entorhinal cortex, among others, has been extensively explored in NDD-related dementia, especially in AD, demonstrating favorable outcomes (Kuhn et al., 2015b; Lozano et al., 2016, 2019).

The NBM is a key component of the basal forebrain (BF), primarily providing cholinergic inputs to the neocortex. It is essential for cognitive function (Gratwicke et al., 2013; Chen et al., 2021) and plays a central role in memory, cortical arousal, attention, and the modulation of cortical plasticity (Kilgard and Merzenich, 1998; Bentley et al., 2004; Kalmbach et al., 2012; Solari and Hangya, 2018). Furthermore, the NBM has been implicated in the pathology of several NDDs, especially AD and PD dementia (PDD) (Liu et al., 2015). Early postmortem studies of the BF identified the presence of significant neuronal loss in the NBM of patients with AD and PD (Whitehouse et al., 1981, 1982; Rogers et al., 1985). This involvement of the NBM in the pathophysiology of NDDs with cognitive impairment suggests that it holds potential as a target for DBS in the treatment of cognitive dysfunction. Although Turnbull et al. (1985) failed to demonstrate definitive cognitive improvement in a 74-year-old patient with AD after 9 months of stimulation, their study provided preliminary evidence of the tolerability of NBM-DBS using a flexible electrode. Subsequently, numerous studies have been conducted to confirm the therapeutic effects of NBM-DBS in various NDDs with cognitive dysfunction, as well as determine the underlying mechanism.

**Figure 1:**
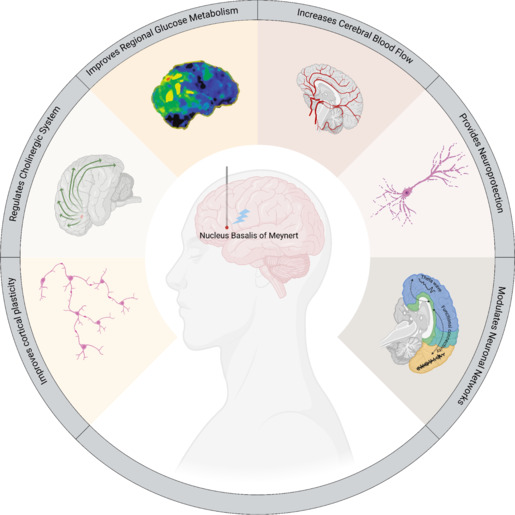
The mechanisms related to deep brain stimulation of nucleus basalis of Meynert. Hypotheses of deep brain stimulation of nucleus basalis of Meynert mechanisms: improving cortical plasticity; regulating the cholinergic system, increasing glucose metabolism and regional cerebral blood flow, providing neuroprotective effects, or modifying neural networks. Created with BioRender.com.

In this review, we first provide an overview of the anatomy and projections of the NBM, along with its physiological role in cognition, its significant involvement in AD and PD, and its connection to cognitive impairment. We next focus on summarizing recent findings from animal studies and clinical researches regarding the potential mechanisms underlying the therapeutic effects of NBM-DBS on cognitive dysfunction in NDDs (**Figure 1**). Finally, we discuss current safety considerations and outline future directions for NBM-DBS.

## Search Strategy

The literature cited in this review were published from 1981 to 2023 and retrieved through an electronic search of Web of Science, and PubMed databases using the following keywords: deep brain stimulation, nucleus basalis of Meynert, basal forebrain, dementia, neurodegenerative diseases, cognition, Alzheimer’s disease, Parkinson’s disease, memory, and cholinergic pathway. After removing irrelevant and duplicate studies from the retrieved research, each article’s title and abstract were read during the preliminary screening. All studies targeting NBM or BF for DBS were included, but studies targeting other DBS sites were excluded.

## Structure and Connectivity of the Nucleus Basalis of Meynert

**Figure 2:**
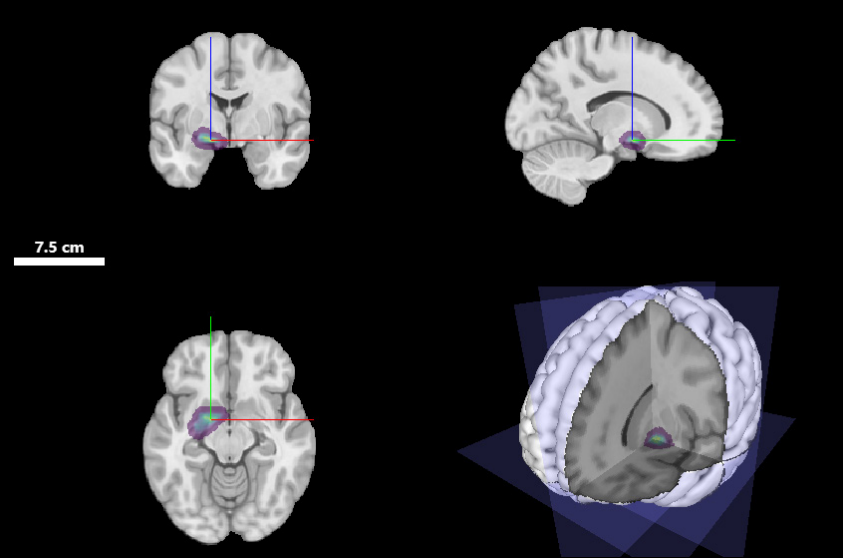
Cellular structure diagram of the human left basal forebrain Ch4 in sagittal, coronal, horizontal, and 3D positions. The colored areas represent the distribution probability of Ch4 cells of basal forebrain, with darker yellow indicating a higher probability. The three colored lines represent the axes of 3D position: the green, red and blue lines respectively indicate the anterior-posterior direction, the medial-lateral direction, and the dorsal-ventral direction. Images sourced from Julich-Brain Atlas, cytoarchitectonic maps (v3.0.3; https://www.julich-brain-atlas.de/; Eickhoff et al., 2005; Evans et al., 2012; Amunts et al., 2020).

Employing histochemical and immunohistochemical labeling of acetylcholinesterase and choline acetyltransferase, Mesulam et al. (1983, 1984) proposed the Ch1–Ch4 nomenclature to describe four cholinergic neuron groups in the BF. The largest of the four groups, Ch4, corresponds to the NBM and is located in the posterior pole of the BF (**Figure 2**). In primates, Ch4 can be further subdivided into three subgroups comprising five subsectors—the anterior part (Ch4a), containing the anteromedial (Ch4am) and anterolateral (Ch4al) subsectors; the intermediate part (Ch4i), consisting of the intermediodorsal (Ch4id) and intermedioventral (Ch4iv) subsectors; and a posterior part (Ch4p) (Boban et al., 2006; Liu et al., 2015). Unlike non-human primates, humans possess a sixth Ch4 subsector, comprising an elongated bridge between the anterior and intermediate part, defined as the anterointermediate (Ch4ai) region (Mesulam and Geula, 1988; Liu et al., 2015).

In humans, the NBM extends horizontally from the olfactory tubercle to the uncal gyrus of the hippocampus and has a length of 13–14 mm in the sagittal plane. The anterior part of the NBM, Ch4a, is limited inferiorly by the horizontal limb of the nucleus of the diagonal band of Broca, supero-medially by the ventral globus pallidus, and supero-laterally by the anterior commissure. The posterior part of the NBM is connected to the ansa lenticularis superiorly, the amygdala inferiorly, the putamen laterally, and the optic tract medially (Mesulam and Geula, 1988; Yu et al., 2019; Chen et al., 2021).

The afferent projections of the NBM are limited to limbic and paralimbic areas and are mostly derived from the central amygdala. Cholinergic, catecholaminergic, and gamma-aminobutyric acidergic (GABAergic) axons comprise the main synaptic inputs to the NBM (Smiley and Mesulam, 1999). The cortical sources of afferent projections consist of the piriform, orbitofrontal, insular, temporopolar, parahippocampal, entorhinal, and cingulate regions. The afferent projections also include subcortical limbic structures such as the amygdala, hypothalamus, septal nuclei, and nucleus accumbens (Jones et al., 1976; Mesulam et al., 1983; Mesulam and Geula, 1988; Gratwicke et al., 2013).

**Figure 3:**
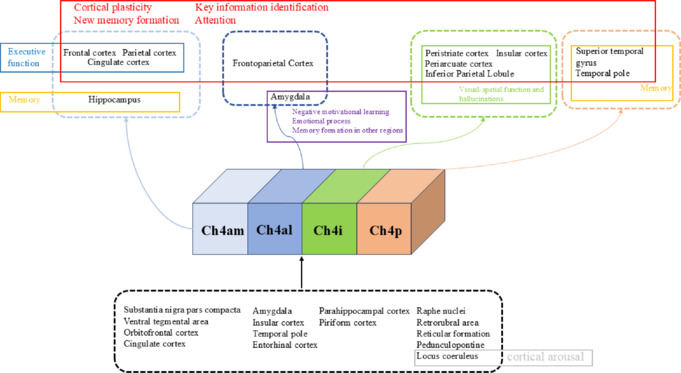
Schematic diagram of major afferent and efferent projections of the NBM and their corresponding functions. The four different colored boxes in the middle represent the four subregions of the NBM; arrows of the same color and the structure names within the dashed boxes indicate the main efferent projection areas for each subregion; the black arrows and the structure names within the dashed boxes represent the main afferent projection areas of the NBM; the solid boxes indicate the functions corresponding to projection areas. NBM: Nucleus basalis of Meynert.

The NBM plays a crucial role in efferent innervation, serving as the primary source of cholinergic projections to the entire cortical surface. The main projection areas of the NBM subdivisions and their potential corresponding physiological functions are described in **Figure 3** (Selden et al., 1998; Gratwicke et al., 2013; Liu et al., 2015; Chen et al., 2021; He et al., 2023). Ch4am supplies the primary cholinergic projection to the frontal, parietal, and cingulate cortices located along the medial wall of the hemisphere. Experiments in rodents, primates, and humans have confirmed that cortical cholinergic function is essential for the acquisition of new memories (Richardson and DeLong, 1988; Croxson et al., 2011). The Ch4al group mainly provides cholinergic projections for frontoparietal opercular regions as well as being the primary source of cholinergic projections to the amygdala. Accordingly, the NBM mediates aversive motivational learning and is known to regulate memory formation in other regions (Richardson and DeLong, 1988; McGaugh, 2002). The Ch4id and Ch4iv groups exhibit similar projection patterns, extending their main projections toward ventrolateral orbital, insular, periarcuate, peristriate, inferotemporal, and parahippocampal regions, in addition to the inferior parietal lobule. The efferent projections of the Ch4p group are limited to the superior temporal gyrus and the temporal pole (Jones et al., 1976; Mesulam et al., 1983; Gratwicke et al., 2013).

Cholinergic fibers originating from the NBM were observed to converge into lateral and medial bundles, which diffusely distribute throughout the cortex. The medial bundle emerges from the anterior part of the NBM, bypasses the posterior part of the corpus callosum, enters the cingulate gyrus, and finally merges with fibers from the lateral bundle before entering the occipital lobe. The lateral bundle has two branches. The fibers of the capsular branch project to the amygdala and temporal cortex after crossing the external capsule, while the perisylvian branch first enters the white matter surrounding the claustrum, and then radiates outward to the frontoparietal cortex, the superior temporal gyrus, and the insula (Selden et al., 1998; Hong and Jang, 2010; Chen et al., 2021).

## Role of the Nucleus Basalis of Meynert in Physiological Cognitive Function

Animal studies indicate that the NBM is a crucial structure for facilitating cognitive and memory functions, effects that are exerted via extensive and intricate fibrous connections to both the cortical and limbic systems. Studies employing excitotoxin infusion or optogenetic and pharmacological methods have found that projections from the NBM to the basolateral amygdala are essential for fear memory and contextual fear extinction memory, while those to the neocortex are vital for the suppression of cued and contextual fear conditioning (Knox, 2016). Additionally, a variety of NBM lesion modalities lead to learning and memory dysfunction. In primates, ibotenic acid-induced NBM lesions led to severe and enduring learning and memory deficits in a visual reversal learning task (Irle and Markowitsch, 1987). Rodents with excitotoxicity-induced lesions in the NBM displayed impaired short-term memory and memory acquisition deficits in the Morris water maze (MWM) test (Mandel et al., 1989). Through the use of a combination of viral transsynaptic tracing systems and choline acetyltransferase-Cre transgenic mice, BF cholinergic neurons (BFCNs) were found to be involved in the regulation of olfaction-related memories. Additionally, subgroups of BFCNs were observed to receive diverse inputs from the olfactory system, forming distinct input patterns, while divergent subgroups of BFCNs were differentially activated in an olfaction-associated learning/memory task (Zheng et al., 2018). Using voltage-sensitive dye imaging, Nagasaka et al. (2017) investigated the functional difference between the anterior (corresponding to Ch4a) and posterior (corresponding to Ch4i) NBM. They found that stimulation of the anterior NBM activated the medial agranular cortical region of the dorsal frontal cortex, which was associated with cognitive function, while stimulation of the posterior NBM activated the frontal cortex more laterally toward the lateral agranular cortex, which was linked to motor function.

The NBM is also involved in the regulation of cortical plasticity and electrophysiological indicators of learning and memory. Pairing an acoustic stimulus with NBM stimulation in adult guinea pigs induced electroencephalogram (EEG) desynchronization and receptive field plasticity comparable to that produced during behavioral learning (Bakin and Weinberger, 1996; McLin et al., 2002). Similarly, compared to each procedure alone, an acoustic stimulus paired with NBM stimulation led to frequency generalization gradients for both cardiac and respiratory behavior in rats, indicating that NBM stimulation paired with tone induced behavioral associative memory (McLin et al., 2002). Another study revealed that an acoustic stimulus paired with NBM stimulation could narrow receptive field sizes, broaden them, or leave them unaltered, depending on the parameters of the auditory stimulus (Kilgard and Merzenich, 1998). Regarding electrophysiology, theta oscillation is associated with episodic memory, such as that involved in spatial navigation, and has long been implicated in learning and memory (Herweg et al., 2020). In mice with bilateral NBM lesions, theta oscillations were shown to be reduced in the hippocampus and increased in the cortex, and these effects were correlated with worse performance in the object recognition and MWM tests (Rispoli et al., 2013). Meanwhile, selective cholinergic lesions in the NBM were reported to produce changes in event-related oscillation energy in theta, beta, and gamma frequency bands and phase lock index in the frontal cortex, central amygdala, and dorsal hippocampus. This suggested that the NBM cholinergic system modified phase synchronization/phase resetting within various brain areas (Sanchez-Alavez et al., 2014).

Through pharmacological manipulations and the selective lesioning of the BF, it was demonstrated that the BFCN system is intimately involved in the mediation of attention. Cholinergic enhancement via the administration of anticholinesterase physostigmine increased the activity of the extrastriate cortex, thereby promoting visual attention, as measured by functional magnetic resonance imaging (fMRI) (Bentley et al., 2004, 2009). In a five-choice serial reaction time task, selective immunotoxic cholinergic lesions in the NBM impaired attention performance (McGaughy et al., 2002; Harati et al., 2008). Lee et al. (2019b) implanted a microelectrode into the NBM of patients with PD and found that the firing characteristics and patterns of NBM neurons changed during an auditory attention-based cognitive task, suggesting that the NBM may be involved in attention-related mechanisms.

## Role of the Nucleus Basalis of Meynert in Alzheimer’s and Parkinson’s Diseases and Evidence of Cognitive Improvement as a Deep Brain Stimulation Target

The NBM has been implicated in the pathology of several NDDs, especially AD and PD (Liu et al., 2015). Early postmortem studies of the BF found that there was substantial neuronal loss in the NBM of patients with these conditions (Whitehouse et al., 1981, 1982; Rogers et al., 1985). The crucial role of the NBM in cognitive function, along with evidence connecting cholinergic damage in the NBM to cognitive impairment in AD and PD, has supported the use of NBM-DBS to address cognitive impairments, resulting in promising initial outcomes (Turnbull et al., 1985; Freund et al., 2009).

### Evidence of abnormalities in the nucleus basalis of Meynert of patients with Alzheimer’s disease

AD is a progressive NDD and the most common cause of dementia, accounting for 60%–80% of all cases of dementia worldwide (Waldemar et al., 2007). Cholinergic neuronal loss, especially in the BF, is a pathological characteristic of AD (Ferreira-Vieira et al., 2016). Whitehouse et al. (1981) reported that up to 90% of NBM cells were lost in postmortem samples of a 74-year-old patient with familial AD who exhibited severe symptoms of dementia before dying. An increasing number of studies have since confirmed the loss of NBM cells in patients with AD based on longitudinal structural magnetic resonance imaging (MRI) and postmortem autopsy (Teipel et al., 2005, 2011; Jethwa et al., 2019; Fernández-Cabello et al., 2020). Immunohistochemical staining of vesicular acetylcholine transporter, choline acetyltransferase, and Aβ in transgenic mouse models of AD has further demonstrated that cholinergic synapses are particularly vulnerable to early Aβ oligomer-mediated neurotoxicity, primarily affecting cholinergic neurons in the NBM (Whitehouse et al., 1981; Ferreira-Vieira et al., 2016). There are different views regarding the location pattern of NBM cell loss. While some studies have identified the anterior NBM as the site exhibiting the most pronounced cell loss (Doucette et al., 1986), others have observed that the posterior sector was more affected (Arendt et al., 1997; Liu et al., 2015). The localization of Ch4p, which provides cholinergic innervation to the temporal pole and superior temporal cortex, in the posterior NBM suggests that the loss of posterior NBM cells correlates well with memory loss and language impairment in AD (Mesulam et al., 1983; Liu et al., 2015). Moreover, postmortem and *in vivo* studies have demonstrated pronounced atrophy of the right NBM in patients with mild cognitive impairment (MCI) and AD (Teipel et al., 2005, 2011), suggesting that the right NBM might be more vulnerable than the NBM to AD pathology. Different neuropathologic subtypes of AD exhibit selective vulnerability in the NBM. There is an association between the severity of neurofibrillary tangle pathology in the NBM and corticolimbic patterns of neurofibrillary tangle pathology in the brain. Younger age at onset was associated with greater neurofibrillary tangle accumulation in the NBM of patients with hippocampal-sparing AD and typical AD, but not limbic-predominant AD (Hanna Al-Shaikh et al., 2020).

Furthermore, the linear thickness of the NBM measured on coronal T1-weighted MRI scans was shown to correlate with cognitive decline and increased levels of cerebrospinal fluid markers of Aβ pathology, and was therefore proposed to be a diagnostic biomarker of AD (Jethwa et al., 2019). Through the calculation of the free-water (FW) value of the locus coeruleus to transentorhinal cortex tract and four magnocellular regions of the BF on diffusion MRI in cognitively normal participants and patients with AD at different pathological stages, it was found that, compared with normal controls, FW was significantly higher in the NBM of AD patients with early MCI. Moreover, the variation in FW values in the NBM was significantly correlated with the severity of cognitive impairment and plasma neurofilament light chain levels in AD patients (Chu et al., 2022). A clinical multimodal MRI study comparing the NBM of patients with AD and normal controls revealed that the former group had smaller gray matter volume and higher regional blood flow in the NBM, along with reduced NBM connectivity with the right insula and cerebellum (Zheng et al., 2020). An increasing number of studies have confirmed that a correlation exists between abnormal patterns of NBM functional connectivity and impaired cognitive domains or responses to cholinesterase inhibitor therapy in patients with AD (Meng et al., 2018; Xu et al., 2021; Lin et al., 2022; Ren et al., 2023). Recently, using Aβ and tau positron emission tomography (PET) and structural MRI, Zeng et al. (2022) found that functional connectivity between the right NBM and the bilateral amygdala increased with the progression of AD pathology. This effect was positively correlated with cerebral amyloidosis, tauopathy, and memory impairment, and was proposed to serve as a potential compensatory mechanism to counteract AD pathology and preserve cognitive function.

Additionally, Schumacher et al. (2021) noted that a reduction in the volume of the NBM was correlated with more severe background slowing on EEG in AD patients with MCI. The same authors subsequently reported that the integrity of the lateral pathway from the NBM was related to global cognition, attentional performance, and EEG background slowing in patients with AD, while a loss of integrity of the medial and lateral NBM pathways was associated with an increased risk of progression to dementia in patients with MCI (Schumacher et al., 2022).

### Role of the nucleus basalis of Meynert in Parkinson’s disease

Cell loss in the NBM was first identified in PD patients in the early 20^th^ century (Liu et al., 2015). This phenomenon is more extensive in PD than in AD, and more apparent among PDD than PD cases (Candy et al., 1983; Perry et al., 1985; Rogers et al., 1985). Substantial evidence supports that the NBM gray matter volume is associated with symptom severity in patients with PDD (Sakai et al., 2019; Pereira et al., 2020; Wilson et al., 2021; Kübler et al., 2022; Rogozinski et al., 2022; Sperling et al., 2023). A reduction in the gray matter volume of the NBM was found to even precede the symptoms of PDD and was indicated to be a predictor for cognitive outcome of bilateral subthalamic DBS in patients with PD (Pereira et al., 2020; Kübler et al., 2022). Similarly, a reduction in NBM volume is associated with apathy, visual hallucinations, delusion, and gait decline in PD (Sakai et al., 2019; Wilson et al., 2021; Sperling et al., 2023). Additionally, the FW fraction of the NBM was found to be correlated with cognitive function in PD patients evaluated using a neuropsychological protocol consisting of language (Boston Naming Test total score, Semantic Fluency total score), visuospatial function (Judgment of Line Orientation), declarative memory (Wechsler Memory Scale-III, Logical Memory Total Recall, Hopkins Verbal Learning Test), cognitive flexibility (Wechsler Adult Intelligence Scale-III Digit Symbol, Letter Number Sequencing, Trail Making Test Part B, and Stroop Color-Word Test), and reasoning (Wechsler Adult Intelligence Scale-III, Matrix Reasoning, Delis-Kaplan Executive function System Tower Test) (Crowley et al., 2022). Rong et al. (2021) also reported that the NBM volume was positively correlated with cortical thickness in the bilateral posterior cingulate, parietal, frontal insular, and left insular regions in PD patients with MCI. Similarly, patients with PD accompanied by NBM atrophy showed significantly reduced metabolism in the parietal and occipital cortices on ^18^F-fluorodeoxyglucose (FDG)-PET (Gang et al., 2020). Investigation of resting state fMRI and seed-based functional connectivity analysis showed that the right NBM of PD patients with MCI exhibited reduced functional connectivity values in the right middle cingulate and paracingulate gyri, middle frontal gyrus, left inferior parietal gyrus, superior frontal gyrus, and other cortical areas compared with those of healthy controls. Additionally, this reduction was positively correlated with cognitive scores in the Mini-Mental State Examination (MMSE), Wechsler Memory Scale, Wechsler Adult Intelligence Scale, animal fluency test from the Montreal Cognitive Assessment, and Hamilton Depression Scale (Zhang et al., 2023).

Lee et al. (2019b) investigated neuronal firing properties in patients with PD-MCI through bilateral globus pallidus pars interna (GPi) and NBM microelectrode implantation. They revealed that neurons in the NBM had significantly lower firing rates and a greater burst index than those of the GPi, while the action potentials of NBM neurons exhibited lower amplitudes and longer repolarization phases relative to GPi neurons. Furthermore, in the oddball task, the firing rates of NBM cells were significantly decreased, whereas those of GPi cells were unaffected. A clinical study employing local field potential (LFP) and EEG recordings in patients with PDD found that delta (1–4 Hz) oscillations were prominently present in the NBM, while cortical coherence was most pronounced in the theta band within temporal areas. Notably, this coherence was virtually absent in the NBM region, possibly due to cholinergic degeneration (Nazmuddin et al., 2018). Rea et al. (2021) revealed that alpha reactivity and pre-alpha power are related to changes in volumes of cholinergic cell clusters corresponding to the posterior NBM in MCI and non-dementia PD patients based on quantitative EEG and assessment of cholinergic BF atrophy. A combination of LFP recordings and cortical magnetoencephalography (MEG) identified three networks that were associated with changes in coherence between the NBM/GPi and the cortex in PD, including a theta (2–8 Hz) band network linking the NBM/GPi and the temporal lobe, a low beta (13–22 Hz) band network linking the NBM/GPi and mesial sensorimotor areas, and a high beta (22–35 Hz) band network connecting the NBM/GPi and lateral sensorimotor areas (Gratwicke et al., 2020a).

## Potential Mechanisms Underlying the Effects of Deep Brain Stimulation of the Nucleus Basalis of Meynert on Cognitive Dysfunction in Neurodegenerative Diseases

### Regulation of the cholinergic system and cortical plasticity

The NBM is a primary component of the BF cholinergic system. Accordingly, an important mechanism underlying the effect of DBS on the NBM in NDDs involves the modulation of the impaired cholinergic system. Acetylcholine is a key neurotransmitter in the central nervous system, playing a major role in cognition and memory (Picciotto et al., 2012; Haam and Yakel, 2017). Through its effects on cholinergic pathways, NBM-DBS can improve attention and alertness, thereby enhancing the ability of patients with NDDs to respond to environmental stimuli as well as ameliorating their cognitive functions. Here, we present a chronological overview of previous work relating to how NBM-DBS regulates the cholinergic pathway.

Early studies demonstrated that DBS of the BF cholinergic system increased acetylcholine release in the cortex of rodents (Rasmusson et al., 1992; Kurosawa et al., 1989a, b), an effect that was found to be frequency-dependent (Rasmusson et al., 1992).

Bakin and Weinberger (1996) investigated the mechanism involved in auditory cortical receptive field plasticity in behavioral learning, a type of physiological memory, in the adult guinea pig. They found that NBM-DBS administered at a current of 270 μA, 200 Hz frequency, specifically increased the responses to conditioned stimuli. This indicated that stimulation of the NBM advances receptive field plasticity and physiological memory in the cortex, and this effect was blocked by systemic and cortical atropine. When combined with auditory cues, NBM-DBS reorganized the representation map of the primary auditory cortex in rodents. However, this regulatory effect on cortical plasticity vanished in animals with highly specific lesions in cholinergic NBM neurons (Kilgard and Merzenich, 1998).

In addition to its association with the regulation of cortical plasticity via the cholinergic pathway, NBM-DBS has been shown to increase nerve growth factor (NGF) release through the acetylcholine nicotinic receptor, further underscoring its close association with the cholinergic pathway (Hotta et al., 2013). Moreover, the sustained subcutaneous infusion of nicotine improved the effect of NBM-DBS on acetylcholine release in rats (Uchida et al., 2011).

In a study on rhesus monkeys, Liu et al. (2017) recently demonstrated that the NBM-DBS-mediated improvement in working memory was related to the levels of acetylcholine and required the activation of acetylcholine receptors, as determined through the administration of cholinesterase inhibitors and acetylcholine receptor blockers. The authors hypothesized that the difference between the effects elicited by intermittent and continuous stimulation could be attributed to reduced total acetylcholine synthesis and release resulting from frequent vesicular release due to continuous stimulation. Interestingly, while treatment with the cholinesterase inhibitor donepezil rescued impairment in performance due to continuous stimulation, it unexpectedly did not further improve performance following intermittent stimulation (Liu et al., 2017). In a subsequent study by Liu et al. (2018), it was shown that improved sustained attention performance induced by NBM-DBS was also connected with the levels of acetylcholine, and could also be impaired by donepezil. These two studies suggested that there might be an optimal level of acetylcholine for performance. Consequently, the combination of NBM-DBS and cholinesterase inhibitor would be ineffective due to excessively high levels of acetylcholine.

In a recent study involving a scopolamine-induced rat model of dementia, intermittent NBM-DBS reportedly increased cholinergic fiber length in the cingulate cortex and the density of synaptophysin immunoreactive presynaptic boutons in the cornu ammonis 1 and dentate gyrus areas (Liu et al., 2022). Unexpectedly, acetylcholine levels in the hippocampus were unaffected after NBM-DBS. The authors suggested that this was due to the lack of direct NBM projections to the hippocampus and that the effect of NBM-DBS on the hippocampus and medial septum was indirect, involving an overall circuit effect. These studies implied that the effect of NBM-DBS on the hippocampus might not directly rely on the levels of acetylcholine. Considering the importance of the hippocampus in memory, further studies are needed to determine how NBM-DBS influences areas such as the hippocampus in the absence of an effect on acetylcholine concentrations.

Notably, continuous stimulation, especially when performed bilaterally, may pose potential risks, resulting in a decrease in cholinergic transmission or leading to more severe adverse events, including mortality (Liu et al., 2017; Huang et al., 2019; Koulousakis et al., 2020). Besides, most research to date on the modulation of the cholinergic pathways is mainly conducted in healthy contexts. There remains a lack of clinical and preclinical studies offering direct evidence on whether NBM-DBS can influence cholinergic pathways in NDDs.

### Increases in regional brain metabolism and blood flow

Glucose is necessary for brain function and tight regulation of its metabolism is crucial for brain activity in both physiological and pathological conditions (Mergenthaler et al., 2013; Hoh Kam and Mitrofanis, 2024). As disease progresses pathologically, AD patients develop brain glucose metabolism disorders and untreated AD patients exhibit an average decline in FDG uptake of 5.2% per year, compared to just 0.9% for healthy controls (Lo et al., 2011). Abnormal glucose metabolism is a core mechanism underlying the development of dementia in both AD and PD and is closely related to cognitive dysfunction and other pathological processes (Chen and Zhong, 2013; Dai et al., 2023). Improving glucose metabolism disorder in patients with AD or PD can contribute to disease treatment and enhance cognitive function (Chen and Zhong, 2013; Dai et al., 2023; Nowell et al., 2023). Additionally, NBM-DBS can enhance cerebral blood flow (CBF) and regional cerebral metabolic rate for glucose (rCMRglc) to support normal neuronal function and metabolic activity, thereby maintaining and improving cognitive abilities.

DBS of the fornix has been demonstrated to promote hippocampal glucose metabolism in aged rats by promoting aerobic respiration (Wang et al., 2018). In addition to animal studies, many clinical investigations have also assessed the effect of DBS on glucose metabolism (Lozano et al., 2016; Jakobs et al., 2020; Teixeira et al., 2022). For instance, in an initial attempt to ameliorate AD symptoms using NBM-DBS, Turnbull et al. (1985) observed that glucose metabolic activity was preserved in the ipsilateral temporal and parietal lobes on the FDG-PET scan relative to that on the contralateral side, although cognitive function did not improve. In a phase I clinical study of NBM-DBS in AD, FDG-PET indicated that glucose utilization in the entire cerebrum and the rCMRglc in the parietal cortex, temporal cortex, and amygdalo-hippocampal region were markedly increased after 1 year of low-frequency DBS (Kuhn et al., 2015b). Recently, Jiang et al. (2022) investigated FDG uptake in six patients with advanced AD who received continuous NBM-DBS. At the 12-month follow-up, FDG uptake was assessed across the salience network (SN), hippocampal network (HIPP), default mode network (DMN), and frontoparietal network (FPN). While FDG uptake did not significantly change in the HIPP, DMN, or FPN, it significantly decreased in the SN. Moreover, a negative correlation was detected between FDG uptake in the HIPP and the Assessment Scale—cognitive subscale (ADAS-cog) score (Jiang et al., 2022).

In contrast, the findings of two recent clinical studies on NBM-DBS did not support the impact of DBS on brain glucose metabolism in other NDDs (Maltête et al., 2021; Sasikumar et al., 2022). In a phase I randomized clinical trial, six patients with Lewy body dementia (LBD) underwent bilateral NBM-DBS at varying frequencies (20, 50, and 100 Hz). Subsequent FDG-PET revealed a notable reduction in metabolic activity in the frontal, parietal, and occipital regions, while a significant increase in metabolic activity was observed in the superior lingual gyrus (Maltête et al., 2021). Meanwhile, in a phase II clinical trial, single-trajectory multiple-targeted DBS was applied to the GPi and the NBM in six patients with advanced PD (Sasikumar et al., 2022). In the NBM-DBS ON condition, FDG-PET indicated that regional glucose metabolism in the right opercular part of the inferior frontal gyrus and the supramarginal gyrus was decreased instead of increased compared to that in the NBM-DBS OFF condition (Sasikumar et al., 2022). Additionally, an early preclinical study utilizing the quantitative [^14^C]2-deoxy-D-glucose method found that NBM-DBS did not affect glucose utilization in the cerebral cortex of anesthetized rats (Kimura et al., 1990). Moreover, in conscious rats, results employing the same technique revealed that NBM-DBS led to only mild variation in cerebral glucose utilization, increasing in the frontal and entorhinal areas and decreasing in the parietal areas ipsilateral to the NBM stimulation relative to that in the contralateral cerebral hemisphere (Lacombe et al., 1997; Vaucher et al., 1997). Conversely, a significant increase in glucose utilization was observed in subcortical regions, particularly in structures of the ipsilateral extrapyramidal system and the NBM. Distinct bilateral decreases were noted in the vestibular and cerebellar nuclei in NBM-stimulated rats when compared to control animals (Vaucher et al., 1997).

Similarly, several early studies utilizing a variety of technologies observed that both electrical and chemical stimulation of the NBM resulted in a widespread increase in CBF across many cortices in rodents (Biesold et al., 1989; Kurosawa et al., 1989b; Adachi et al., 1990a, b; Hallström et al., 1990). In rats, focal electrical stimulation or the microinjection of L-glutamate in the NBM increased CBF in the parietal lobe anesthetized with carbamate (Biesold et al., 1989). Furthermore, the vasodilatory response occurred only ipsilateral to the stimulation site.

Subsequent studies found that changes in regional CBF resulting from NBM-DBS were localized to areas receiving cholinergic nerve projections from the NBM (Adachi et al., 1990a, b). The sustained subcutaneous infusion of nicotine further enhanced NBM-DBS-induced cortical vasodilation, confirming that this effect of DBS primarily depended on the cholinergic pathway (Uchida et al., 2011). Cholinergic fibers emanating from the NBM terminate in the cortex in a layer-specific manner (Luiten et al., 1985). Accordingly, Hotta et al. (2013) measured changes in the diameters of penetrating arteries in different layers of the cortex during NBM stimulation using two-photon microscopy. Their results indicated that the diameters of the cortical penetrating arteries were increased in a depth-dependent manner, which was consistent with the layer specificity of the cholinergic projections from the NBM to the cortex.

To determine if age is a contributing factor to cortical CBF following NBM-DBS, Kurosawa et al. (1989b) compared the CBF in the parietal cortex between healthy adult (6–8 months old) and aged (27–28 months old) anesthetized Fisher-344 rats after NBM-DBS, and observed no significant difference between the two groups. In contrast, Lacombe et al. (1997) found that NBM-DBS increased frontal and parietal CBF in young rats (2–4 months old), while this incremental effect was reduced by half in aged rats (22–28 months old). The enhancement of cortical CBF seems beneficial to neurons. In a rat model of transient ischemia, the NBM-DBS-mediated increase in CBF delayed cortical neuronal death due to ischemia; however, it remains unclear whether this improvement is beneficial to pathological changes in neurodegeneration (Hotta et al., 2002).

These results illustrated that the effect of NBM-DBS on CBF in the cortex primarily relies on the function of cholinergic projections that can generate the vasodilative response, with metabolism playing a minimal role. Studies in which the rCMRglc, extracellular lactate levels and systemic arterial blood pressure were simultaneously measured have suggested that variations in CBF within the cortex are not linked to metabolism or arterial blood pressure. In contrast, a coupling between CBF and rCMRglc was observed in subcortical areas (Lacombe et al., 1997; Vaucher et al., 1997). This discrepancy highlights the complex mechanisms underlying the impact of NBM-DBS in different brain regions and further suggests that the alterations in metabolism evoked by NBM-DBS are region-dependent. Further research is needed to explore the regional specificity and mechanisms of NBM-DBS in the regulation of brain metabolism, as well as whether its effects on CBF and rCMRglc are related.

Most clinical studies are structured as Phase I trials focusing on assessing the safety of NBM-DBS in a limited number of patients and do not possess adequate power to evaluate the efficacy of NBM-DBS in enhancing glucose metabolism. Additionally, the variability in disease severity and lesion distribution among participants may influence the outcomes of NBM-DBS. Variations in stimulation patterns and parameters, along with differences in causes and conditions of patients with NDDs, might account for the discrepancies in these studies. Furthermore, small sample sizes can result in significant variability in statistical analysis results. More preclinical studies and larger clinical cohorts, combined with a more unified scale and coincident stimulation patterns, are urgently needed to explore the specific role of NBM-DBS in regional glucose metabolism and blood flow in the brain.

### Neuroprotective effects

It is widely acknowledged that NGF plays an important role in neuronal development, providing neuroprotection and facilitating neural repair through multiple mechanisms (Sofroniew et al., 2001; Wu et al., 2024). In the BF cholinergic system, NGF is essential for the maintenance of BFCN phenotypes and can ameliorate cholinergic neuron loss in aged rats (Fischer et al., 1987; Cuello et al., 2007). Evidence suggests that increasing NGF levels can exert neuroprotective effects on cholinergic neurons (Aloe et al., 2015). In patients with AD, the targeted delivery of the NGF gene into the forebrain improved the rate of cognitive decline as assessed by ADAS-cog and MMSE, and increased cortical rCMRglc levels (Tuszynski et al., 2005). NBM-DBS may enhance cognitive function in patients with NDDs by elevating NGF levels, activating anti-apoptotic pathways in neurons, and improving neuronal survival.

The neuroprotective effect of NBM-DBS may be linked to NGF. Hotta et al. (2007) demonstrated that unilateral NBM-DBS increased ipsilateral cortical extracellular NGF concentrations in anesthetized healthy adult rats (4–5 months old). Further investigation revealed that unilateral NBM-DBS in adult rats (4–6 months old) resulted in an increase in extracellular NGF levels in the ipsilateral parietal cortex, whereas no similar changes were observed in aged rats (29–31 months old) (Hotta et al., 2009). In addition, mecamylamine, a nicotinic receptor antagonist, eliminated this increase, suggesting that this effect of NBM-DBS may be related to nicotinic receptor activation (Hotta et al., 2009). Notably, the increase in NGF levels reduced the production of Aβ in patients with AD and transgenic mouse models of the condition (Triaca et al., 2016). These findings suggest that NBM-DBS improves the survival of neurons in different brain regions by upregulating NGF levels and further enhancing cognitive function (Koulousakis et al., 2020; Liu et al., 2022).

NBM-DBS may exert therapeutic effects by promoting neuron survival, as evidenced by studies on DBS targeting other brain regions (Temel et al., 2006; Wallace et al., 2007). An early study investigating the activation effects of NBM-DBS in the adult rat brain found that, compared to controls, rats subjected to NBM-DBS exhibited a greater number of c-Fos-labeled cells in the prefrontal cortex, dorsal cornu ammonis, and ventral dentate gyrus (Boix-Trelis et al., 2006). The neuroprotective effect of NBM-DBS may also be achieved by altering apoptosis-related gene expression, thereby decreasing the rate of apoptosis and improving neuronal survival (Chen et al., 2014; Meng et al., 2016). Recently, Liu et al. (2022) observed that NBM-DBS increased the number of BrdU/NeuN double-labeled cells (a marker of newborn neurons) in the dentate gyrus and the expression of c-Fos in multiple regions related to memory, such as subregions of the hippocampus and the entorhinal cortex, a constituent of the Papez circuit. Similarly, NBM-DBS decreased ischemia-induced apoptosis in neurons. Additionally, Huang et al. (2019) noted that NBM-DBS reduced neuronal apoptosis in the hippocampus and cortex of APP/PS1 mice (murine model of AD). The detection of apoptosis-related proteins identified significant decreases in the levels of caspase-3, caspase-8, and Bid, indicating that NBM-DBS may increase neuronal survival through an anti-apoptotic effect. The authors further found that the phosphoinositide 3-kinase/protein kinase B (PI3K/AKT) pathway was upregulated while the extracellular signal-regulated kinase (ERK) 1 and 2 pathway was downregulated. Both pathways are mainly involved in cell proliferation and growth, and significantly influence cell survival, synaptic plasticity, and memory function. These properties render them plausible therapeutic targets for NDDs and have been assessed for their neuroprotective effects in these conditions (Goyal et al., 2023; Indrigo et al., 2023). It has also been demonstrated that the function of NGF can exert its effects through activation of the PI3K/AKT pathway (Kim et al., 2014), which potentially explains that the effect of NBM-DBS on activating the PI3K/AKT pathway in AD mice could be related to release of NGF (Huang et al., 2019).

Despite research demonstrating that NBM-DBS effectively promotes NGF release, no evidence exists regarding its impact on NGF levels in disease states. It is currently unknown whether NBM-DBS can promote NGF release in the context of reduced NGF levels in neurodegenerative conditions such as AD and PD. Furthermore, more detailed research is needed to confirm the relationship between NBM-DBS-induced NGF release and the improvement of cognitive function in NDDs.

### Modification and reconstruction of damaged neural networks

The NBM, a part of the BF, serves as the primary source of cholinergic innervation to the neocortex. NBM-DBS can modulate the firing patterns of neurons targeted by its projections, thus modifying related neural networks (Gratwicke et al., 2013). NBM-DBS may operate at the brain network level to synchronize activity across multiple brain regions, consequently enhancing cognitive function.

Single-pulse 100 Hz NBM-DBS in rats evoked a depolarizing response in frontal neural activity as determined by voltage-sensitive dye imaging. Additionally, stimulation of the anterior and posterior NBM elicited a regional discrepancy in neural activity within the frontal cortex, corresponding to the medial and lateral agranular cortex, respectively (Nagasaka et al., 2017). In primates, Qi et al. (2021) observed that intermittent NBM-DBS (15–20 s/min) improved the performance of adult monkeys in a working memory task and increased neural activity in the dorsolateral prefrontal cortex, aligning with the effects of cholinergic agonists. Unlike cholinergic agonists, however, NBM-DBS led to broader tuning of individual neurons in the prefrontal cortex, stabilizing the network that maintains information in working memory. Both studies (Nagasaka et al., 2017; Qi et al., 2021) illustrated that NBM-DBS can alter the discharge pattern of the neocortex to enhance working memory in monkeys.

Furthermore, to confirm the effect of NBM-DBS on global neural activity, Singh et al. (2022) analyzed LFPs in the frontal cortex of two healthy monkeys. In rodents, GABAergic projections from the BF to the cortex are associated with gamma-band oscillations and are responsible for the rhythmic entrainment of cortical neurons, while NBM-DBS increases neural activity in the prefrontal cortex. The authors hypothesized that the effect of NBM-DBS on cortical neurons might be exerted via the activation of ascending GABAergic projections, which would be reflected in an increase in gamma band power (McKenna et al., 2013; Kim et al., 2015; Záborszky et al., 2018; Qi et al., 2021). Unexpectedly, compared to baseline, there was no significant difference in gamma power during the delay period of the working memory task after NBM-DBS, whereas a prominent reduction in alpha power was observed at all stages of the delay interval of the task (Singh et al., 2022). The decreased effect of NBM-DBS on alpha power during the delay period of the task suggested that the network was less likely to shift attention to the second stimulus, thereby stabilizing the information in memory and improving working memory performance (Singh et al., 2022). Also in primates, Liu et al. (2017) implanted stimulation electrodes in the NBM and recorded the LFPs. They observed that both 20-second intermittent NBM-DBS at 60 Hz and continuous stimulation at 80 Hz elicited a consistent decrease in the LFP power spectrum (frequency bands including < 4 Hz, 4–8 Hz, 8–12 Hz, 12–20 Hz, and 20–40 Hz) relative to the pre-stimulation level.

In a clinical study on AD, low-frequency NBM-DBS maintained EEG power in the theta, alpha, and beta bands over 1 year, whereas a decline was observed with drug therapy alone (Kuhn et al., 2015a). In PD patients with MCI, a MEG study indicated that in the NBM-DBS ON state, low-frequency activity (delta and/or theta) was increased in the left frontal, parietal, and temporal lobe regions, while high-frequency activity (beta and/or low gamma) was increased more posteriorly, including in the right occipital and cerebellar regions, compared with the OFF state (Sasikumar et al., 2022). In addition, the same study revealed that NBM-DBS increased coherence within left-hemisphere regions of interest (including left frontal, parietal, occipital, and cerebellar delta, theta, and low-gamma bands). Inter-hemispheric coherence increased between left and right frontal (delta band), left and right sensorimotor (delta, beta, and low-gamma bands), and left and right cerebellar (delta band) regions, while an improvement in intra-hemispheric coherence was detected between the right frontal and right temporal areas (theta and alpha bands) (Sasikumar et al., 2022). These changes in oscillations partially reversed the background slowing typical of PDD and increased cortico-cortical coherence in all frequency bands, possibly counteracting the loss of cortico-cortical connections associated with cognitive decline in PD (Bosboom et al., 2006, 2009; Gratwicke et al., 2020a; Maltête et al., 2021). Despite these positive results, the cognitive function of the patients did not show any improvement. The authors attributed the discrepancy between neuroimaging findings and cognitive outcomes to variations in the sensitivity of these measures, low statistical power, and the sample size.

Changes in human neural networks have been shown to differ among various NDDs (Seeley et al., 2009). In advanced PD, it has been demonstrated that changes in the DMN on fMRI correspond to the extent of cognitive decline (Spetsieris et al., 2015; Colloby et al., 2016). To determine whether NBM-DBS could modulate a disrupted DMN, Gratwicke et al. measured resting-state fMRI of patients with PDD under low-frequency NBM-DBS, and found no significant difference between active and sham DBS groups (Gratwicke et al., 2018). However, in a subsequent study involving patients with LBD, it was found that NBM-DBS elicited bidirectional changes in functional connectivity in resting state fMRI, consisting of a reduction in functional connectivity between the posterior cingulate cortex and the right inferior parietal cortex and an increase in functional connectivity among the intra-parietal sulcus, inferior frontal gyrus, and superior parietal lobule (Gratwicke et al., 2020b). Furthermore, by combining LFP and MEG recordings, the authors identified the presence of patterns of long-range cortical synchrony both in the NBM and GPi in patients with LBD or PDD after NBM-DBS. Three networks were revealed in both groups of patients after NBM-DBS, including a theta (2–8 Hz) band network between the NBM/GPi and temporal lobe, a low beta (13–22 Hz) band network between the NBM/GPi and mesial sensorimotor areas, and a high beta (22–35 Hz) band network between the NBM/GPi and lateral sensorimotor areas (Gratwicke et al., 2020a). Compared to individuals diagnosed with LBD, those with PDD exhibited significantly higher beta oscillatory power in the GPi region. Additionally, compared to the PDD group, a coherence pattern in the high beta band between the GPi region and the lateral sensorimotor cortex exhibited greater consistency across patients and hemispheres in the LBD group (Gratwicke et al., 2020a). In a subsequent study, Oswal et al. (2021) utilized a joint MEG and LFP analysis to explore the intersection of structural and functional connectivity in six PDD and five LBD patients after undergoing NBM-DBS. Three distinct NBM-cortical networks with overlapping structural and functional connectivity were found, including a low beta band network (13–22 Hz) between the NBM and the Supplementary Motor Area, driven by the cortex; a delta/theta band network to medial temporal lobe structures encompassing the parahippocampal gyrus; and a delta/theta band network to visual areas, including the lingual gyrus (Oswal et al., 2021). However, it remained unknown whether these networks were affected by NBM-DBS, given that pre-DBS condition measurements were not included as a control.

Recently, a clinical study relating to NBM-DBS in patients with advanced AD investigated the connections among four networks—the SN, HIPP, DMN, and FPN. During the NBM-DBS ON period, functional connectivity between the HIPP and FPN tended to increase, as did that between the SN and FPN, the SN and HIPP, and the DMN and FPN. Seed-based functional connectivity analysis among 14 regions in the four networks illustrated that NBM-DBS modulated the functional connectivity of multiple networks. Correlation analysis of these modulated networks found that changes in parahippocampal gyri/insula connectivity were negatively associated with the ADAS-cog score (Jiang et al., 2022). Furthermore, three patterns of state of structured functional connectivity were identified, thus revealing the dynamic functional connectivity changes occurring during NBM-DBS. The three states occurred in similar proportions in the NBM-DBS OFF period at the 1-month follow-up, where state 1 exhibited more frequent and robust connections, state 2 displayed relatively sparse connections, and state 3 was characterized by strong connections specifically linking the DMN and the FPN. Compared to the DBS-OFF state, the proportion of state 2 significantly increased during the NBM-DBS ON period, which was positively correlated with the rate of reduction of the MMSE score and negatively correlated with the rate of increase of the ADAS-cog score (Jiang et al., 2022).

Although some effects of NBM-DBS on brain networks in NDDs have been revealed, they represent only a small part relative to the complexity of brain networks under pathological conditions. Additionally, NBM-DBS may improve impaired neural networks only in limited circumstances, and its effectiveness is very restricted in patients with advanced stages of disease. Although the timing of DBS is crucial to its effectiveness (Tan et al., 2020), data on early intervention is scarce. This may preclude a comprehensive understanding of the potential benefits of NBM-DBS, as several studies have indicated that it exerts more favorable effects in younger patients and those in the early stages of the disease (Kuhn et al., 2015a; Baldermann et al., 2018; Dürschmid et al., 2020). It is essential to better understand the specific relationships between neural networks and NBM-DBS to clarify both the effect of NBM-DBS on cognition in NDDs and the underlying mechanisms. Additionally, specific effects of NBM-DBS on neural networks and electrophysiological indicators could serve as biomarkers, offering valuable guidance for optimizing the DBS strategy and enabling real-time, responsive, closed-loop DBS (Senova et al., 2018).

## Therapeutic Effect of Deep Brain Stimulation of the Nucleus Basalis of Meynert on Cognition

### Animal studies on deep brain stimulation of the nucleus basalis of Meynert: the effect on cognition

The therapeutic effects of NBM-DBS on cognition have been widely investigated in animals, as detailed in **Additional Table 1**. In a 192 IgG-saporin-induced rat model of AD, bipolar stimulation with the parameters 120 Hz, 90 μs, and 1 V improved spatial memory performance as assessed by the MWM test (Lee et al., 2016). A separate investigation sought to determine the optimal stimulation parameters for NBM-DBS in APP/PS1 transgenic mice at various ages (Huang et al., 2019). This study confirmed the effect of NBM-DBS in restoring spatial memory in the MWM test and further found that 100 Hz stimulation lasting 21 days at 4 months of age produced the most significant enhancement of spatial memory performance. A comparable study in a scopolamine-induced rat model of dementia assessed the impact of NBM-DBS on cognitive function using various stimulation parameters (Liu et al., 2022). In contrast to Huang et al. (2019), low-frequency intermittent stimulation with the parameters of 20 Hz, 100 μA, and a biphasic pulse shape led to a significant enhancement of recognition memory performance, as assessed using object location and object recognition tasks.

**Additional Table 1:** Animal studies of NBM-DBS for the treatment of cognitive impairment

Study	Species/sample size	Age	Disease model	NBM coordinates	Stimulation paradigm	Main results on cognitive function	Safety or adverse events
Lee et al., 2016	Rats/25	6 wk	192 IgG- saporin induced BFCN degeneration	Right NBM: AP: -1.32 mm ML: +2.8 mm DV: -7.4 mm relating to Bregma	Bipolar Unilateral 120 Hz, 90 μs, 1 V, 1 h/d, persisting 7 d	Behavioral performance of MWM was improved to a normal level	Despite the electrodes being positioned near the pallidum and internal capsule, implanting them and stimulating the NBM resulted in no adverse effects, such as seizures or abnormal behavior
Liu et al., 2017	Monkeys/5	Adult	Healthy	NBM: AP: 16 mm anterior interaural ML: ±8 mm lateral DV: 29 mm below the cortical surface	Condition 1:1200 pulses per minute were delivered in 10, 20, 40, or 60 s. Condition 2: 80 pluses per second with different durations per minute, varied to 10, 20, 40, or 60 s.	Continuous stimulation of NBM at 80 Hz during a particular interval of the task impaired the performance of monkeys in a delayed match-to-sample task. In condition 1, monkeys with 10 or 20 s stimulation per minute of NBM and in condition 2, monkeys with 20 s stimulation per minute showed a better performance in the task than 60 s continuous stimulation	NBM-DBS provides the same therapeutic benefits as high-dose donepezil, without the nausea side effects caused by donepezil. The result suggests that continuous stimulation may lead to depletion of acetylcholine reserves
Liu et al., 2018	Monkeys/2	6 yr	Healthy	NBM: AP: 16 mm anterior interaural ML: ±8 mm lateral DV: 29 mm below the cortical surface	Biphasic pulse train Initial negative phase of 200 mA, followed by a positive phase of 200 mA, and lasting for 100 ms per phase 60 Hz, 20 s ON, 40 s OFF	NBM-DBS increased the sustained attention reflecting in the increased hit rates and decreased false rates of monkeys in the modified continuous performance task	NA
Huang et al., 2019	Mice/192	10 wk-12 mon	APP/PS1 transgenic AD model	Left NBM: AP: -0.7 mm ML: +1.75 mm DV: -4.0 mm relating to Bregma	Biphasic pulse wave Unilateral Different frequencies: 10, 50, 100, 130 Hz 90 μs, 1 A 1 h/d Different durations: 7, 14, 21, 28 d	NBM-NBS at the optimized parameters (100 Hz, 90 μs, 1 A, 1 h/d, persisting 21 d, starting at 4 mon age) improved the performance of APP/PS1 transgenic AD model mice in MWM, which was partially attenuated but did not counteract by U0126 and LY2940020	A preliminary experiment demonstrated that bilateral NBM-DBS resulted in more severe complications and increased mortality
Koulousakis et al., 2020	Rats/12	18 mon	TgF344 transgenic AD model	Bilateral NBM: AP: -1.44 mm ML: ±2.88 mm DV: -7.4 mm relating to Bregma	Positive monopolar pulses 100 μs, 200 μA Intermittent stimulation: 60 Hz, 20 s ON, 40 s OFF Continuous stimulation: 20 Hz	Both unilateral and bilateral intermittent stimulation of NBM improved the performance of AD mice in modified Barnes maze task, while continuous stimulation had no effect. There was no significant difference in discrimination index in NOR among unilateral intermittent stimulation, bilateral intermittent stimulation and continuous stimulation groups	Five animals developed a kindling effect upon continuous stimulation
Qi et al., 2021	Monkeys/2	Adult	Healthy	Right or left NBM: AP: 16 mm anterior interaural ML: ±8 mm lateral DV: 29 mm below the cortical surface	Biphasic Negative first Unipolar 200 mA pulses with 100 ms per phase 80 Hz pulses were delivered for 100 ms Intermittent stimulation 80 pulses per second, 15 s ON, 45 s OFF	Intermittent stimulation of NBM improved the working memory performance of monkeys in the remember-first and remember-second tasks	NA
Liu et al., 2022	Rats/22	Adult	Scopolamine-induced dementia model	Bilateral NBM: AP: -1.3 mm ML: ±2.8 mm DV: -7.4 mm relating to Bregma	MONOPHASIC (100 ps pulse width) or biphasic pulse shape (280 s, 80 μs zero interval between a 100 μs positive pulse and a 100 ps negative pulse) Low and high frequency (20 or 120 Hz) 100 μA amplitude Different durations: continuous or intermittent (20 s ON, 40 s OFF)	20 Hz, biphasic pulse shape, 100 μA amplitude at 20 s ON, 40 s OFF stimulation of NBM significantly restored scopolamine-induced memory loss in OLT and ORT, while there was no difference between DBS and sham groups in OFT and elevated zero maze	In the OFT and the EZM, there was no clear evidence indicating that NBM-DBS causes anxiety- related side effects

AD: Alzheimer's disease; AP: anterior-posterior; APP/PS1: amyloid-β precursor protein/Presenilinl; BFCN: basal forebrain cholinergic neurons; DBS: deep brain stimulation; DV: dorsal-ventral; EZM: elevated zero maze; LY294002: a phosphoinositide 3-kinase inhibitor; ML: medial-lateral; MWM: Morris water maze; NA: not available or mentioned in the reference; NBM: nucleus basalis of Meynert; NOR: novel object recognition task; OFT: open field test; OLT: object location task; ORT: object recognition task; U0126: an extracellular signal-regulated kinases 1 and 2 inhibitor.

In non-human primates, Liu et al. (2017) demonstrated that intermittent stimulation of the NBM (60 pulses per second for 20 seconds every minute) improved working memory performance in a delayed match-to-sample task. Conversely, continuous stimulation impaired working memory performance, an effect that could be reversed by donepezil, suggesting that continuous stimulation might affect acetylcholine release. In addition, measurements taken 5 months after NBM-DBS illustrated that its effects on working memory were long-lasting. A subsequent study demonstrated that intermittent stimulation improved the performance of monkeys in a continuous performance task, indicating that intermittent stimulation played an auxiliary role in sustaining attention (Liu et al., 2018).

Conclusions regarding the parameters and patterns of NBM-DBS in animals remain inconsistent. In these studies on rodents, there was no significant difference in the pulse width or current intensity of the stimulation, which were approximately 90–100 μs and 100–200 μA, respectively (Lee et al., 2016; Huang et al., 2019; Koulousakis et al., 2020; Liu et al., 2022). However, the frequency of stimulation varied. In two studies, high-frequency stimulation at 120 and 100 Hz was shown to enhance cognitive impairment in cholinergic-deficient and transgenic AD models, as assessed by the MWM test (Lee et al., 2016; Huang et al., 2019). Additionally, the latter study demonstrated that stimulation at 50 and 130 Hz effectively enhanced cognition in AD model mice, whereas stimulation at 10 Hz had no effect (Huang et al., 2019). In this study, stimulation was applied for one hour using constant pulse waves.

In contrast, in the other two studies, continuous pulse stimulation did not lead to significant cognitive improvement, although evaluation was performed differently, that is, using the novel object recognition task (Koulousakis et al., 2020; Liu et al., 2022). In the second study, only biphasic intermittent stimulation at 20 Hz (40 seconds ON, 20 seconds OFF) elicited a clear beneficial effect on cognition. No combination of 120 Hz, monophasic or biphasic pulse shapes, whether continuous or intermittent, resulted in significant cognitive improvement (Liu et al., 2022). Although differences in the animal models (scopolamine-induced dementia model *versus* transgene AD model) and evaluation methods (novel object recognition task versus the MWM test) between the two studies may have contributed to the divergent results, further experiments are necessary to clarify the reasons for these opposing findings (Huang et al., 2019; Liu et al., 2022). The primate studies, conducted by the same research team, examined the effects of various stimulation parameters on healthy adult monkeys. They discovered that continuous stimulation at 80 Hz negatively impacted the monkeys’ working memory performance. Subsequently, they tested two modes of intermittent stimulation, one delivering 1200 pulses per minute over durations of 10, 20, 40, or 60 seconds and another with 80 pulses per second, varying in duration from 10, 20, 40, or 60 seconds per minute. Stimulation for 20 seconds in both modes resulted in the best working memory performance (Liu et al., 2017, 2018; Qi et al., 2021).

### Clinical studies on deep brain stimulation of the nucleus basalis of Meynert in neurodegenerative diseases

The clinical studies examining the therapeutic effects of NBM-DBS in NDDs are summarized in **Additional Table 2**. In an attempt to rescue the remaining cholinergic projections from the NBM to the cortex and enhance the cortical metabolic activity in a patient with AD, Turnbull et al. (1985) first implanted a flexible electrode into the left NBM. After 8 months of NBM-DBS, no significant changes in the cognitive evaluation scores were detected. However, given the encouraging evidence for the positive effects of NBM-DBS on cognitive function, clinical trials of NBM-DBS in patients with AD were subsequently initiated. In a phase I study, six patients with mild to moderate AD, aged between 57 and 79 years, were recruited. After 1 year of bilateral NBM-DBS (20 Hz, 90 μs, 2.5 V), the ADAS-cog and MMSE scores of these patients showed slower deterioration compared with those of patients treated with a cholinesterase inhibitor (Gillette-Guyonnet et al., 2011; Kuhn et al., 2015b). Furthermore, NBM-DBS was conducted on two younger patients with milder AD, and, over a long-term, 24-month follow-up of all eight patients, the two younger patients in the earlier stages of the disease showed greater benefits from NBM-DBS, as evaluated by ADAS-cog (Kuhn et al., 2015a; Hardenacke et al., 2016). Another clinical trial of NBM-DBS in AD used a low-frequency parameter to investigate whether the extent of preoperative brain atrophy served as an outcome predictor for NBM-DBS in cognition and memory. A correlation was identified between fronto-parieto-temporal patterns of cortical thickness and improved clinical results as assessed by ADAS-cog and MMSE (Baldermann et al., 2018). Through the observation of EEG components in a passive auditory oddball paradigm, Dürschmid et al. (2020) revealed that NBM-DBS reinstated mismatch negativity, a prominent EEG component most likely signaling prediction errors, to a normal level and had a beneficial effect on the recognition of familiar tonal stimuli compared to the DBS-off state. Zhang et al. (2021) reported that monopolar stimulation of the NBM at the parameters of 20 Hz, 90 μs, and 1 V elicited an improvement in ADAS-cog and MMSE scores in an 80-year-old male with advanced AD. A study by the same group investigated the modulatory effect of NBM-DBS in eight patients with advanced AD. Continuous NBM-DBS at 20 Hz was observed to stabilize ADAS-cog and MMSE scores at the 1-year follow-up (Jiang et al., 2022).

**Additional Table 2:** Clinical studies of NBM-DBS for the treatment of cognitive impairment

Study	Sample size/age (yr)	Diagnosis	NBM coordinates	Stimulation parameters	Main results on cognitive function	Safety or adverse events
Turnbull et al., 1985	1/74	AD	Left NBM X = 8 mm^a^ *Y* = 11 mm *Z* = 5 mm	3.0 V, 210 ms, 50 Hz 15 s ON, 12 min OFF	Failed to identify any response to stimulation clinically after 9-mon NBM-DBS	NBM-DBS for 8 mon did not lead to epilepsy or any other negative effects
Freund et al., 2009	1/71	PDD	Bilateral NBM and STN X = 12.5 mm^b^ *Y* = 4 mm *Z* = 5 mm	1.0 V, 120 μs, 20 Hz	Bilateral stimulation of the NBM improved the cognitive function in Auditory Verbal Learning Test, Clock Drawing Task and Trail Making Test Part A	NA
Kuhn et al., 2015b	6/57-79	AD	Bilateral NBM X = 20.0-33.2 mm^b^ *Y* = 6.2-10.0 mm Z = 5.2-10 mm	2.0-4.5 V, 90-150 μs, 10-20 Hz	There was an increase of 3 points in the ADAS-cog score and a decrease of 0.5 points in the mean MMSE score after 12-mon NBM-DBS. The mean score of the Clinical Dementia Rating remained stable over the 12-mon follow-up.	Two significant adverse events, both related to hardware, resulted from a failure of the plug-in connector. One patient reported experiencing inner restlessness at elevated stimulation intensities (> 5 V)
Kuhn et al., 2015a	2/61 and 67	AD	NA	20 Hz	In the ADAS-cog, patient 1 deteriorated by 7 points after 26 mon, but patient 2 did not change after 28 mon. In MMSE, patient 1 deteriorated by 2 points after 26 mon, whereas patient 2 improved by 2 points after 28 mon.	NA
Hardenacke et al., 2016	8/NA	AD	NA	NA	The MMSE scores of four out of the eight patients remained relatively consistent throughout the 24-mon follow-up.	NA
Gratwicke et al., 2018	6/35-80	PDD	Bilateral NBM X = 17.6-23.0 mm^c^ *Y* = 4.9-9.5 mm *Z* = 2.9-6.4 mm	1.5-3.0 V, 60 μs, 20 Hz	After 6-mon stimulation, there was only an improvement in NPI total score. No significant improvement was observed in any of the scales in the cognitive function including California Verbal Learning Test-II, Wechsler Adult Intelligence Scale-III, Delis-Kaplan Executive Function System, Posner covert attention test, Cambridge Neuropsychological Test Automated Battery, reaction time test, MMSE and MDRS	No serious adverse events were reported during the trial period. One patient experienced scalp electrode cap erosion 15 mon after the trial commenced
Baldermann et al., 2018	10/61-75	AD	NA	2.0-4.2 V, 60-150 μs, 5-20 Hz	In the follow-up of 6 and 12 mon, the mean MMSE score decreased by 0.4 points and improved by 1.8 points respectively, the ADAS-cog mean score increased by 1.6 points and 2.3 points, respectively. And the Alzheimer's Disease Assessment Scale Memory Subscale mean score decreased by 0.4 points and 3.1 points, respectively	NA
Nombela et al., 2019	1/68	PD with MCI	Bilateral GPi and NBM* X = 24.20 mm^c^ *Y* = 3.71 mm *Z* = 5.10 mm	2 mA, 60 μs, 20 Hz	Except for limited deterioration observed in the MMSE, the subscale conceptualization from MDRS-II, and the Judgment of Line Orientation test (less than 10% worsening each), as well as a 1-point reduction in the Reverse Digit Span subscale, the other majority of the neuropsychological tests showed improvements after combined GPi and NBM stimulation, compared to single GPi stimulation	No visual side effects were noted during the follow-up
Dürschmid et al., 2020	8 (6 healthy controls)/59-63	AD	NA	1 V, 20 Hz	In the condition of DBS OFF, the mismatch negativity significantly differed from controls and this difference disappeared when NBM-DBS turned on. Patients with NBM-DBS ON (but not with NBM-DBS OFF) and controls perform more attenuated responses to frequently repeated standard tones	NA
Gratwicke et al., 2020b	6/50-80	LBD	Bilateral NBM X = 16.8-21.4 mm^c^ *Y* = 4.2-11.5 mm Z = 4.2-8.0 mm	2.0-3.5 V, 60 μs, 20 Hz	After 6-mon stimulation, NBM-DBS decreased the median NPI score from 15 to 9 points. There was no significant change in either abbreviated cognitive battery or the additional assessments	One patient developed colitis associated with antibiotics. Two patients exhibited slight increases in confusion and paranoia right after electrode implantation surgery
Zhang et al., 2021	1/80	AD	Bilateral NBM X = 20.3 mm (right), 23.5 mm^d^ *Y* = 3.6 mm (right), 6.2 mm (left) *Z* = 6.2 mm (right), 6.5 mm (left)	1.5 V, 90 μs, 20 Hz	After 10-wk NBM-DBS, compared to baseline, ADAS-cog was improved from 43 to 33 points. Furthermore, MMSE score was improved from 5 to 9 point	The patient developed bilateral perioperative subdural effusions following wire implantation. After increasing the stimulation voltage, the patient experienced mood swings, loss of appetite, and abnormal eating behaviors
Maltête et al., 2021	6/50-69	LBD	Bilateral NBM ** X = 18.7-24.2 mm *Y* = 18.2-22.4 mm *Z* = 1.8-6.1 mm	2-3 V, 60-90 μs, 20-100 Hz	Compared to sham DBS, there was no significant improvement in cognition after 3 mon of NBM-DBS evaluated by Free and Cued Selective Reminding Test, MMSE, FAB, MDRS, Stroop test, Verbal fluency test, Benton total score, TMT, Wisconsin Card Sorting Test score, Wechsler Intelligence Scale for Children cubes score, Rey figure score, Praxic abilities, DO80 score, NPI total score and Epworth Sleepiness Scale score	All patients tolerated the surgery well, and there were no adverse events associated with the procedure. One patient exhibited postoperative delirium linked to a urinary tract infection. Eleven non-serious adverse events were reported in four patients, including falls, low back pain, confusion, and others
Cappon et al., 2022	6/35-80	PDD	Bilateral NBM X = 17.6-22.2 mm^c^ *Y* = 4.9-9.5 mm *Z* = 2.9-6.4 mm	3.0 V, 60 μs, 20 Hz	Over 36-mon follow-up, the mean median score of MMSE and DRS-2 decreased from 24 to 17 and 116 to 84.5, respectively. The change between individual patients of MMSE and DRS-2 varied markedly. Similarly, there are differences among individual patients in NPI, Blessed Dementia Scale, Zarit Burden Interview and Short Form 36 Health Survey. There is a significant correlation between the baseline scores of Posner covert attention test and Scales for Outcomes in Parkinson's Disease sleepiness scale and lost points of MMSE and DRS-2	The monitoring of adverse events indicated that no serious adverse events took place during the 36-mon follow-up period
Sasikumar et al., 2022	6/61-70	PD with MCI	Bilateral GPi and NBM ** X = 20.0 mm^c^ *Y =* 5.0-8.0 mm *Z* = 5.0 mm	2.5-8.5 mA, 5-60 μs, 15- Hz	LFS of the NBM, instead of acute HFS improved 60 the accuracy on the sustained attention task during the programming session relative to the OFF condition. After a 1-yr follow-up, the cognitive functions of two patients remained stable, while the other four patients showed a cognitive decline, as evidenced by the progression from PD-MCI to PDD and further deterioration across various measures encompassing multiple cognitive domains. There was no improvement in neuropsychological scores at the individual level, and no statistically significant changes were observed on any measure at the group level	NBM DBS was well tolerated
Jiang et al., 2022	8/59-78	AD	Bilateral NBM X = 20.7-28.5 mm^e^ *Y* = 4.7-10.1 mm *Z* = 5.2-10.1 mm	2.0-3.0 V, 90 μs, 20 Hz	The MMSE and ADAS-cog scores did not change compared to baseline at the 12-mon follow-up. However, after NBM-DBS for 1 mon, the MMSE score of these patients showed a significant improvement	The surgery was well-tolerated by all patients, and none experienced adverse neurological effects

a X is the distance behind the anterior commissure, Y is the distance lateral to midline and Z is the distance below the intercommissural line. b X is the distance lateral to the third ventricle wall, Y is the distance posterior to the anterior border of the posterior commissure, and Z is the distance ventral to the anterior commissure-posterior commissure line. c Stereotactic coordinates reference to the midcommissural point of the anterior commissure-posterior commissure plane. d X is the distance lateral to the midline, Y is the distance posterior to the anterior commissure and Z is the distance ventral to the anterior commissure. e X is the distance lateral, Y is the distance posterior to the anterior commissure, Z is the distance inferior to the anterior commissure-posterior commissure line. * The reference for the coordinates is not clearly specified. ** This coordinate is GPi and the electrode advances 2.4 mm to reach NBM after detecting GPi. AD: Alzheimer's disease; ADAS-cog: Alzheimer's Disease Assessment Scale-Cognitive Subscale; DRS: Dementia Rating Scale; GPi: globus pallidus internus; HFS: high frequency stimulation; LBD: Lewy body dementia; LFS: low frequency stimulation; MCI: mild cognitive impairment; MDRS: Mattis Dementia Rating Scale; MMSE mini mental status examination; NA: not available or mentioned in the reference; NBM: nucleus basalis of Meynert; NPI: neuropsychiatric inventory; PDD: Parkinson's disease dementia; STN: subthalamic nucleus.

Similarly, the impact of NBM-DBS was tested in patients with PD. A patient with progressive Parkinson-dementia syndrome underwent electrode implantation in the bilateral subthalamic nucleus (STN) and the NBM, aiming at separately treating motor and cognitive functions. This provided the first confirmation of the therapeutic effect of NBM-DBS on cognition (Freund et al., 2009). Single bilateral STN-DBS (right side 4.2 V, 130 Hz, 60 μs; left side 3.5 V, 130 Hz, 60 μs) improved motor symptoms, but did not affect memory performance. Notably, significant improvements in cognitive function were observed with combined stimulation of the bilateral STN (right side 4.2 V, 130 Hz, 60 μs; left side 3.5 V, 130 Hz, 60 μs) and NBM (right side 1.0 V, 20 Hz, 120 μs; left side 1.0 V, 20 Hz, 120 μs). Single NBM-DBS was not performed as the patient refused to accept the consequences of STN-DBS being turned off. However, bilateral low-frequency (20 Hz) stimulation of the NBM in six patients with PDD did not yield significant improvements in cognition (Gratwicke et al., 2018). Additionally, during a follow-up of six PDD patients who received 6 weeks of NBM-DBS over 36 months, three patients experienced a rapid decline and progression of dementia, while one patient died. However, two patients exhibited stabilized cognitive function as measured by the MMSE and Dementia Rating Scale-2, which typically decline in patients with PDD (Cappon et al., 2022). In a study involving the simultaneous stimulation of the GPi and the NBM in PDD, cognitive enhancement was detected, as evidenced by the scores in most of the neuropsychological tests performed, except for minor deterioration noted in the MMSE, the conceptualization subscale from Mattis Dementia Rating Scale-II, and the Judgment of Line Orientation test, along with a 1-point reduction in the Reverse Digit Span subscale after 3 months of combined stimulation (Nombela et al., 2019). Recently, a phase II double-blind, randomized, crossover pilot trial involving patients with PDD reported that while simultaneous high-frequency stimulation of the GPi and low-frequency stimulation of the NBM ameliorated motor complications, it exerted a lesser effect on cognitive function (Sasikumar et al., 2022).

The impact of NBM-DBS on cognitive impairment has also been examined in LBD. Gratwicke et al. (2020b) noted that NBM-DBS improved neuropsychiatric symptoms in patients with LBD as evaluated by the Neuropsychiatric Inventory. Another study on NBM-DBS in LBD, using frequencies ranging from 20 to 100 Hz, found no significant differences in the Free and Cued Selective Reminding Test and the MMSE scores between the NBM-DBS and the sham DBS groups, whereas fluctuations in cognitive functions were improved, as measured using the One Day Fluctuation Assessment Scale (Maltête et al., 2021). The authors attributed this lack of improvement to the advanced stage of the disease and the brief duration of the stimulation.

Evidence from current studies indicates that the therapeutic effect of NBM-DBS on cognition is more pronounced in patients with AD than in those with PDD or LBD, in which the effect remains uncertain. A common limitation of these studies is that small cohorts reduce the credibility of the results. Additionally, discrepancies may arise from variations in parameters, patterns, stimulation duration, and disease stage. Ultimately, younger patients or those with better cognitive conditions may benefit more from NBM-DBS therapy. This finding aligns with the results obtained in transgenic mouse models of AD (Huang et al., 2019). A comprehensive study with a uniform stimulus pattern and a larger sample size is essential to confirm the clinical benefits of NBM-DBS on cognitive dysfunction in NDDs.

All current studies on NBM-DBS in AD and PD and a comparison of the impact of NBM-DBS on these two conditions are summarized in **Additional Table 3**.

**Additional Table 3:** Comparison of the role of NBM-DBS in AD and PD

Impact aspects	AD	PD
Glucose metabolism		
FDG-PET	NBM-DBS preserved glucose metabolic activity in the ipsilateral temporal and parietal lobes (Turnbull et al., 1985). NBM-DBS increased the glucose utilization of entire cerebrum, parietal cortex, temporal cortex and amygdalo-hippocampal region (Kuhn et al., 2015b). There was no significant decrease in FDG uptake in HIPP, DMN and FPN, while a significant decrease of FDG uptake was observed in SN (Jiang et al., 2022).	NBM-DBS decreased regional glucose metabolism in the right opercular part of the inferior frontal gyrus and the supramarginal gyrus (Sasikumar et al., 2022).
Neural networks		
EEG	NBM-DBS maintained the EEG power of theta, alpha, and beta band over 1 yr, in contrast to a decline in AD with sole drug therapy (Kuhn et al., 2015b). In the condition of DBS OFF, the mismatch negativity significantly differed from controls and this difference disappeared when NBM-DBS turned on (Dürschmid et al., 2020).	NA
MEG	NA	NBM-DBS ON state displayed increased low-frequency activity (delta and/ or theta) in the left frontal, parietal, and temporal lobe regions, while high- frequency activity (beta and/or low gamma) increased more posteriorly, including in the right occipital and cerebellar regions (Sasikumar et al., 2022).
fMRI	During the NBM-DBS ON period, functional connectivity between the HIPP and FPN tended to increase, as did the connectivity of SN/FPN, SN/HIPP and DMN/FPN (Jiang et al., 2022).	The resting-state fMRI of PDD patients under low-frequency NBM-DBS displayed no significant difference between active and sham DBS groups (Gratwicke et al., 2020b).
Cognitive changes		
Animal behavior tests	NBM-DBS improved the performance of APP/PS1 transgenic AD model mice in MWM (Huang et al., 2019). Both unilateral and bilateral intermittent stimulation of NBM improved the performance of AD mice in modified Barnes maze task, while continuous stimulation had no effect (Koulousakis et al., 2020).	NA
Clinical assessment	Failed to identify any response to stimulation clinically after 9 mon NBM-DBS (Turnbull et al., 1985). There was an increase of 3 points in the ADAS-cog score and a decrease of 0.5 points in the mean MMSE score after 12-mon NBM-DBS. The mean score of the Clinical Dementia Rating remained stable over the 12-mon follow-up (Kuhn et al., 2015b). In the ADAS-cog, patient 1 deteriorated by 7 points after 26 mon, but patient 2 did not change after 28 mon. In MMSE, patient 1 deteriorated by 2 points after 26 mon, whereas patient 2 improved by 2 points after 28 mon (Kuhn et al., 2015a). The MMSE scores of four out of the eight patients remained relatively consistent throughout the 24-mon follow-up (Hardenacke et al., 2016). In the follow-up of 6 and 12 mon, the mean MMSE score decreased by 0.4 points and improved by 1.8 points, respectively, the ADAS-cog mean score increased by 1.6 points and 2.3 points, respectively. And the Alzheimer's Disease Assessment Scale Memory Subscale mean score decreased by 0.4 points and 3.1 points, respectively (Baldermann et al., 2018). After 10-wk NBM-DBS, compared to baseline, ADAS-cog was improved from 43 to 33 points. Furthermore, MMSE score was improved from 5 to 9 points (Zhang et al., 2021). The MMSE and ADAS-cog scores did not change compared to baseline at the 12-mon follow-up. However, after NBM-DBS for 1 mon, the MMSE score of these patients showed a significant improvement (Jiang et al., 2022).	Bilateral stimulation of the NBM improved the cognitive function in Auditory Verbal Learning Test, Clock Drawing Task and Trail Making Test Part A (Freund et al., 2009). After 6 mon stimulation, there was only an improvement in NPI total score. No significant improvement was observed in any of the scales in the cognitive function including MMSE, Wechsler Adult Intelligence Scale-III and so on (Gratwicke et al., 2018). Except for limited deterioration observed in the MMSE, the subscale conceptualization from MDRS-II, and the Judgment of Line Orientation test (less than 10% worsening each), as well as a 1-point reduction in the Reverse Digit Span subscale, the other majority of the neuropsychological tests showed improvements after combined GPi and NBM stimulation, compared to single GPi stimulation (Nombela et al., 2019). Over 36 mon follow-up, the mean median score of MMSE and DRS-2 decreased from 24 to 17 and 116 to 84.5 respectively (Cappon et al., 2022). After a 1-yr follow-up, the cognitive functions of two patients remained stable, while the other four patients showed cognitive decline, as evidenced by the progression from PD-MCI to PDD. There was no improvement in neuropsychological scores at the individual level, and no statistically significant changes were observed on any measure at the group level (Sasikumar et al., 2022).

AD: Alzheimer's disease; ADAS-cog: Alzheimer's Disease Assessment Scale-Cognitive Subscale; DBS: deep brain stimulation; DRS: Dementia Rating Scale; DMN: default mode network; EEG: electroencephalogram; FDG-PET: 18F-fluorodeoxyglucose-positron emission tomography; fMRI: functional magnetic resonance imaging; FPN: frontoparietal network; GPi: globus pallidus internus; HIPP: hippocampal network; MCI: mild cognitive impairment; MDRS: Mattis Dementia Rating Scale; MEG: magnetoencephalography; MMSE mini mental status examination; MWM: Morris water maze; NA: not available or mentioned in the reference; NBM: nucleus basalis of Meynert; NPI: neuropsychiatric inventory; PD: Parkinson's disease; PDD: Parkinson's disease dementia; SN: salience network.

## Safety of Deep Brain Stimulation of the Nucleus Basalis of Meynert

According to studies published to date, NBM-DBS is generally regarded as relatively safe. Its safety profile and adverse effects are summarized in **Additional Tables 1** and **2**. The side effects of NBM-DBS are currently not a major concern in animal studies. The safety profile of NBM-DBS appears to be relatively acceptable based on several studies that have reported adverse events (Lee et al., 2016; Liu et al., 2017, 2022; Huang et al., 2019; Koulousakis et al., 2020). However, some potential issues need to be addressed through further research.

Continuous stimulation might present potential risks. Liu et al. (2017) discovered that continuous stimulation at 80 Hz negatively affected working memory performance in monkeys, and this effect was reversed by donepezil. Similarly, Koulousakis et al. (2020) found that 5 out of 12 TgF344 transgenic AD model mice exhibited a kindling effect following continuous stimulation. Importantly, in these animals, continuous stimulation was administered bilaterally. In another study utilizing unilateral NBM-DBS to treat AD model mice, preliminary experiments indicated that bilateral NBM-DBS resulted in severe complications and increased mortality rates, although detailed information was lacking (Huang et al., 2019).

Several clinical studies have evaluated the technical feasibility and safety of NBM-DBS (Kuhn et al., 2015b; Gratwicke et al., 2018; Zhang et al., 2021). In the treatment of AD patients, Kuhn et al. reported that one patient experienced inner restlessness at NBM-DBS intensities > 5 V (Kuhn et al., 2015b). Despite this, neural stimulation itself did not lead to any adverse events. In another case report of NBM-DBS in patients with AD, as the voltage was gradually increased, the patient’s body temperature rose from 36.5 to 37.7°C, accompanied by mood fluctuations, loss of appetite, and abnormal eating behaviors (Zhang et al., 2021). The authors suggested two possible reasons for the increase in body temperature. In cases of severe brain atrophy, the distance between the NBM and the hypothalamus decreases, increasing the likelihood of abnormal stimulatory effects on the latter. Alternatively, an increase in acetylcholine levels due to NBM-DBS may activate cold sensors, resulting in changes in body temperature. In other reports relating to the therapeutic effect of NBM-DBS treatment on AD, it is generally noted that there are no significant adverse events, and patients demonstrate good tolerance to the treatment.

Generally, NBM-DBS seems to be both feasible and safe for use in the treatment of PDD. Gratwicke et al. reported that low-frequency 20 Hz NBM stimulation in six affected patients was safe and well-tolerated, except for one patient who required surgical removal of the right electrode cap due to scalp erosion. Notably, the surgical procedure had no adverse effects on cognition (Gratwicke et al., 2018). No serious adverse events occurred during the 36-month follow-up, in line with another study involving six patients with PDD who underwent NBM-DBS (Cappon et al., 2022).

In two studies on NBM-DBS treatment for LBD, adverse events were reported in patients, including mild confusion, paranoia, and falls (Gratwicke et al., 2020b; Maltête et al., 2021). However, these were not attributed to the stimulation itself, and all patients tolerated the surgery well.

Current research suggests that carefully selected patients with cognitive and psychiatric symptoms can consent to and tolerate this treatment without experiencing serious adverse events or cognitive decline.

## Future Perspectives

The exploration of NBM-DBS is currently in its early stages, and more research is needed to refine treatment strategies. Considering the significant individual differences in current studies, larger study cohorts are needed to explore its effectiveness and to identify the characteristics of patient populations that respond more effectively to NBM-DBS. Similarly, future research should include more younger patients in the early disease stages, as the ineffectiveness in late-stage patients might obscure the potential effects of NBM-DBS. Additionally, the current stimulation methods used in clinical applications are limited. Developing more optimal individualized stimulation patterns could help enhance the effectiveness of NBM-DBS, such as responsive closed-loop stimulation. In this context, future research should focus on exploring biomarkers for NBM-DBS treatment of cognitive impairments, developing intelligent and real-time responsive DBS devices, that can adapt to rapidly shifting neural networks at different stages of NDDs with cognitive dysfunction. Similarly, it is of great interest to combine NBM-DBS with precise neurosystem-targeted drugs to explore multidementional therapy options to promote more comprehensive neural network recovery in NDDs with impaired cognition.

## Conclusion

**Figure 4:**
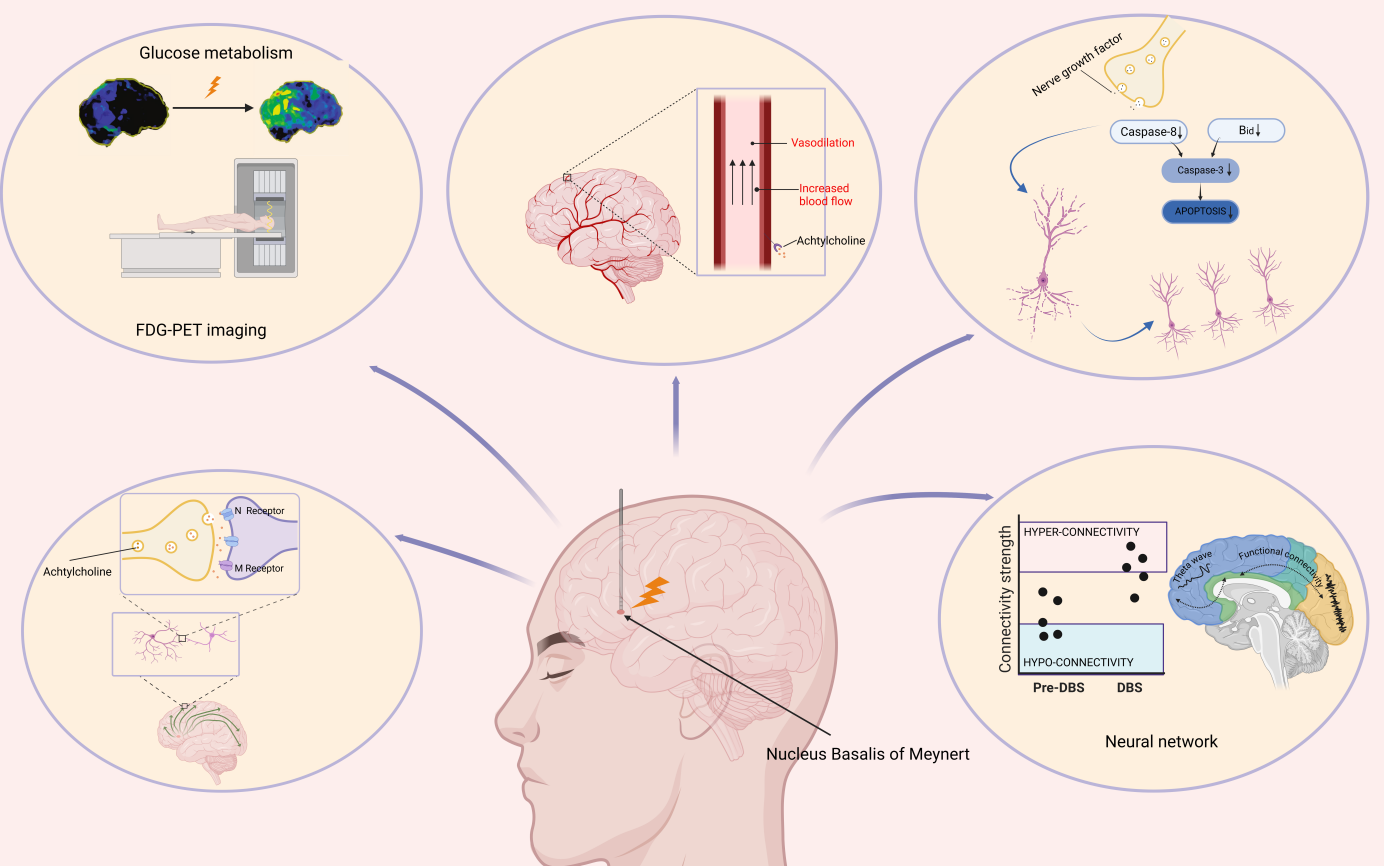
Potential treatment mechanisms of NBM-DBS in NDDs with cognitive dysfunction. Middle bottom is the schematic diagram of NBM-DBS in humans. Bottom left shows that NBM-DBS modulates the cholinergic pathway and promotes the release of acetylcholine in the projection areas. Top left shows that NBM-DBS modulates regional glucose metabolism as revealed by FDG-PET. Middle top shows that NBM-DBS regulates regional cerebral blood flow and modulates arterial vessel diameter, and this effect is associated with acetylcholine release. Top right shows that NBM-DBS rescues degenerating neurons in NDDs by promoting nerve growth factor release and downregulating apoptosis-related genes, thereby enhancing neuronal survival and providing neuroprotective effects. Bottom right shows that NBM-DBS modifies and reconstructs the damaged neural networks in NDDs through electroencephalogram and functional connectivity assessment, enhancing the connectivity between networks. Created with BioRender.com. DBS: Deep brain stimulation; FDG-PET: ^18^F-fluorodeoxyglucose-positron emission tomography; NBM: nucleus basalis of Meynert; NDDs: neurodegenerative diseases.

Currently, there exist no extraordinarily helpful strategies for the treatment of dementia in NDDs, such as PDD and AD, to prevent or reverse disease progression. As a neuromodulation surgery targeting cognition related neural structures and networks, NBM-DBS has been applied to alleviate cognitive impairments in NDDs. Clinical studies have explored the potential of NBM-DBS to improve cognitive impairments in NDDs (Freund et al., 2009; Kuhn et al., 2015a; Hardenacke et al., 2016; Nombela et al., 2019; Dürschmid et al., 2020; Zhang et al., 2021; Jiang et al., 2022). Similarly, in animal researches, NBM-DBS has been demonstrated to improve behavioral performance in domains such as spatial memory, working memory, attention, and other aspects (Lee et al., 2016; Liu et al., 2017, 2018, 2022; Huang et al., 2019; Koulousakis et al., 2020; Qi et al., 2021). Moreover, the potential mechanisms of NBM-DBS in treating NDDs with cognitive dysfunction have been revealed (**Figure 4**), including enhancing the release of acetylcholine in the cortex, regulating glucose metabolism, increasing the CBF, improving NGF release in cortex, altering the apoptosis related gene expression to improve the survival of neurons and modifying and reconstructing the damaged neural networks.

In addition, limitations still existed. Almost all studies have small sample sizes and mostly involve elderly patients in the late stages of the disease. Additionally, most studies use stimulation parameters derived from previous research, and the effectiveness of these parameters has not been fully validated. Currently, basic research on the mechanisms of NBM-DBS is still in its early stages, lacking more deeper and detailed studies. Larger-scale, multi-center trials at earlier stages of the disease are necessary for clinical studies to fully explore optimal parameters. Meanwhile, mechanistic studies necessitate a comprehensive and systematic multimodal approach to thoroughly explain the mechanisms of NBM-DBS, thereby suggesting improved responsive closed-loop stimulation parameters and patterns are urgently needed.

## Additional files:

***Additional Table 1:*** *Animal studies of NBM-DBS for the treatment of cognitive impairment.*

***Additional Table 2:*** *Clinical studies of NBM-DBS for the treatment of cognitive impairment.*

***Additional Table 3:*** *Comparison of the role of NBM-DBS in AD and PD.*

## Data Availability

*All relevant data are within the paper and its Additional files*.
